# Peptidoglycan-Binding Anchor Is a *Pseudomonas aeruginosa* OmpA Family Lipoprotein With Importance for Outer Membrane Vesicles, Biofilms, and the Periplasmic Shape

**DOI:** 10.3389/fmicb.2021.639582

**Published:** 2021-02-25

**Authors:** Magnus Paulsson, Kasper Nørskov Kragh, Yu-Ching Su, Linda Sandblad, Birendra Singh, Thomas Bjarnsholt, Kristian Riesbeck

**Affiliations:** ^1^Clinical Microbiology, Department of Translational Medicine, Faculty of Medicine, Lund University, Malmö, Sweden; ^2^Division for Infectious Diseases, Skåne University Hospital, Lund, Sweden; ^3^Faculty of Health and Medical Sciences, Costerton Biofilm Center, University of Copenhagen, Copenhagen, Denmark; ^4^Department of Integrative Medical Biology, Umeå University, Umeå, Sweden; ^5^Department of Clinical Microbiology, Copenhagen University Hospital, Copenhagen, Denmark

**Keywords:** biofilm, lipoproteins, OMV, outer membrane vesicles, peptidoglycan, *Pseudomonas aeruginosa*

## Abstract

The outer membrane protein A (OmpA) family contains an evolutionary conserved domain that links the outer membrane in Gram-negative bacteria to the semi-rigid peptidoglycan (PG) layer. The clinically significant pathogen *Pseudomonas aeruginosa* carries several OmpA family proteins (OprF, OprL, PA0833, and PA1048) that share the PG-binding domain. These proteins are important for cell morphology, membrane stability, and biofilm and outer membrane vesicle (OMV) formation. In addition to other OmpAs, *in silico* analysis revealed that the putative outer membrane protein (OMP) with gene locus PA1041 is a lipoprotein with an OmpA domain and, hence, is a potential virulence factor. This study aimed to evaluate PA1041 as a PG-binding protein and describe its effect on the phenotype. Clinical strains were confirmed to contain the lipoprotein resulting from PA1041 expression with Western blot, and PG binding was verified in enzyme-linked immunosorbent assay (ELISA). By using a Sepharose bead-based ELISA, we found that the lipoprotein binds to *meso*-diaminopimelic acid (mDAP), an amino acid in the pentapeptide portion of PGs. The reference strain PAO1 and the corresponding transposon mutant PW2884 devoid of the lipoprotein were examined for phenotypic changes. Transmission electron microscopy revealed enlarged periplasm spaces near the cellular poles in the mutant. In addition, we observed an increased release of OMV, which could be confirmed by nanoparticle tracking analysis. Importantly, mutants without the lipoprotein produced a thick, but loose and unorganized, biofilm in flow cells. In conclusion, the lipoprotein from gene locus PA1041 tethers the outer membrane to the PG layer, and mutants are viable, but display severe phenotypic changes including disordered biofilm formation. Based upon the phenotype of the *P. aeruginosa* PW2884 mutant and the function of the protein, we designate the lipoprotein with locus tag PA1041 as “peptidoglycan-binding anchor” (Pba).

## Introduction

*Pseudomonas aeruginosa* is a Gram-negative opportunistic pathogen that can adapt to a wide range of environments by means of its unusually large genome (Stover et al., 2000). It can cause various human infections, including acute and chronic infections of the lungs, keratitis, wound infections, urinary tract infections, and bacteremia. It poses a particular threat for patients in critical care, where *P. aeruginosa* is one of the most important causes of ventilator-associated pneumonia, and for patients with cystic fibrosis (CF) who often acquire persistent infections causing permanent damage to the lungs (Ciofu et al., 2013; Rello et al., 2014). Moreover, *P. aeruginosa* infections are often difficult to treat because of the wide-ranging antibiotic resistance and the formation of biofilms. Because of this, the World Health Organization has emphasized the critical need for research and development towards new treatment strategies for carbapenem-resistant *P. aeruginosa* (World Health Organization, 2017).

The ability of *P. aeruginosa* to cause chronic infections is partly attributed to the formation of biofilms. Biofilms are aggregations of bacteria surrounded by a matrix containing proteins, DNA, polysaccharides, and parts of the bacterial cell wall (Costerton et al., 1999; Flemming and Wingender, 2010). During *P. aeruginosa* infections of the respiratory tract, non-attached mucus-embedded biofilms are frequently occurring in the bronchi, and attached bacterial biofilms are common in the endotracheal tubes (Bjarnsholt et al., 2009). Abundant formation of biofilms impedes antibiotic treatment against *P. aeruginosa* (Høiby et al., 1977). The biofilm protects bacteria from antibiotic drugs by including bacteria at different growth stages and obstructing drug diffusion into the inner cells of the matrix (Evans et al., 1991; Walters et al., 2003).

The cell wall of Gram-negative bacteria consists of a lipopolysaccharide (LPS)-covered outer membrane (OM) and an inner phospholipid bilayer membrane that surrounds the periplasmic space. The peptidoglycan (PG) layer is contained within the periplasmic space. The PG layer gives the bacterial cell stability through its elastic semi-rigid structure of linear glycan strands and cross-linked pentapeptides (Koch and Woeste, 1992). The outer membrane proteins (OMPs) and the lipoproteins associated with the outer membrane protein A (OmpA) family attach to the PG layer with a conserved domain at the C-terminus, while the N-terminus is attached to the outer membrane. The PG attachment has been characterized in greatest depth in *Escherichia coli* and *Acinetobacter baumannii*, where the OmpA homologs were found to bind diaminopimelic acid (DAP), an amino acid present only in PG pentapeptide: L-Ala-γ-D-Glu-*meso*-DAP-D-Ala-D-Ala of Gram-negative bacteria. OmpA homologs from different species are highly similar in the C-termini and are predicted to share similar PG-binding characteristics. Similar domains are present in, for instance, *P. aeruginosa* OprF, OprL, PA0833, and PA1048, *E. coli* OmpA and Pal, *A. baumannii* AbOmpA, *Haemophilus influenzae* P6, and *Klebsiella pneumoniae* KpOmpA (Bouveret et al., 1999; Parsons et al., 2006; Park et al., 2012; Yang et al., 2018).

The N-termini of OmpA homologs are less conserved than the PG-binding C-termini and differ markedly between OmpA family proteins. OprF, OmpA, and P6 have been reported as highly multifunctional proteins and are required for bacterial virulence. OmpA proteins are involved in interactions between bacteria and the human host at several levels during infection, including the innate immune system (complement system) and adhesion to eukaryotic cells, as well as interactions to neighboring bacterial cells in biofilms. The OmpA family proteins are highly immunogenic, and several OmpAs are or have been evaluated as potential vaccine targets (Prasadarao, 2002; Prasadarao et al., 2002; Fito-Boncompte et al., 2011; Bouffartigues et al., 2015; Mishra et al., 2015).

Disruption of the OmpA–PG attachment has been suggested to cause increased shedding of nanosized spherical OMVs from the outer membrane, although more elaborate theories involving *Pseudomonas* quinolone signal (PQS) have questioned this (Wessel et al., 2013). *Pseudomonas aeruginosa* growing as planktonic cells and in biofilms release OMVs, which either diffuse away from the cells or form part of the biofilm matrix (Schooling and Beveridge, 2006). The OMVs are composed of cell wall structures including lipids, LPS, and membrane proteins, but also periplasmic and cytoplasmic contents, such as DNA (Beveridge, 1999; Hellman et al., 2002; Badmasti et al., 2015). They provoke a severe inflammatory response, and their presence is integral in the host–pathogen interaction during pneumonia (Paulsson et al., 2018).

Locus PA1041 in the *P. aeruginosa* PAO1 genome encodes for a hypothetical lipoprotein that shares the conserved C-terminal with other OmpA family proteins. In the present study, we aimed to confirm the PA1041 gene product as a PG-binding protein in the outer membrane of *P. aeruginosa.* To specify the binding site, recombinantly expressed lipoproteins and purified PGs, as well as components of the PG, were included in the analyses. We present experimental evidence showing that the lipoprotein derived from PA1041 is expressed in four clinical strains. In addition, we examined cell wall-related phenotypic and morphologic traits that are known to be important for virulence. A mutated *P. aeruginosa* lacking the lipoprotein formed a biofilm with increased thickness but of low density and shed excessive amounts of OMVs. Based upon these observations, we propose that the protein encoded by locus PA1041 is to be designated as “peptidoglycan-binding anchor” (Pba) with the gene name *pba*.

## Materials and Methods

### Bacterial Strains and Growth Conditions

The bacterial strains used in this study are listed in Table 1. Clinical *P. aeruginosa* strains were kindly supplied by the Clinical Microbiology, Laboratory Medicine Skåne (Lund, Sweden), and species identities were confirmed with MALDI-TOF (Bruker Daltonics, Bremen, Germany). The transposon insertion mutant PW2884 with *lac*Z insert in the gene locus PA1041 was acquired (Two-Allele Library, University of Washington, Seattle, WA) (Held et al., 2012). Bacteria were cultured at 37°C on blood agar plates, lysogeny broth (LB) agar plates or in liquid broth, M9 minimal medium with 0.3 mM glucose, or Jensen medium, as indicated. The growth rates were plotted as a function of the absorbance measured every 5 min at OD_600_ in a microplate spectrophotometer (Fluostar Omega; BMG Labtech, Ortenberg, Germany). Cultures containing *E. coli* with expression vectors were supplemented with appropriate antibiotics as indicated.

**TABLE 1:** Clinical *Pseudomonas aeruginosa* isolates, laboratory strains, and plasmids used in this study.

**Name**	**Description/genotype**	**References**
**Clinical isolates**		
PA KR794	Urine isolate	Paulsson et al., 2015
PA KR796	Airway isolate from a patient with CF	Paulsson et al., 2015
PA KR799	Blood isolate	Paulsson et al., 2015
PA KR801	Airway isolate from a patient with CF	Paulsson et al., 2015
**Laboratory strains**		
*E. coli* BL21(DE3)	*E. coli* laboratory strain	Studier and Moffatt, 1986
*E. coli* DH5α	*E. coli* laboratory strain	Taylor et al., 1993
*E. coli* pET16b-*pba*	*E. coli* BL21(DE3) for soluble expression of Pba	This study
*E. coli* pET16b-*oprG*	*E. coli* BL21(DE3) for soluble expression of OprG	Paulsson et al., 2019
PAO1	*P. aeruginosa* reference strain	Held et al., 2012
PW2884	PAO1 mutant with ISlacZ/hah transposon insert in *pba*	Held et al., 2012
PW2884 + *pba*	PW2884 complemented with pUCP22Not-*pba*	This study
**Plasmids**		
pUCP22Not-*pba*	Complementation plasmid with PA1041 insert. Gentamicin^*r*^	Herrero et al., 1990
pET16b-*pba*	6 × His-tagged Pba expression vector. Ampicillin^*r*^	This study
pMRP9	GFP expression vector. Carbenicillin^*r*^	Davies et al., 1998

### DNA Cloning and Protein Expression

The open reading frame (ORF) *pba* was amplified from the genomic DNA of *P. aeruginosa* PAO1 using primers 5′-CTGAGGATCCGGCAGGGTTGCAGAAAAGCGACTGGC-3′ and 5′-CTGACAAGCTTTTCGCGCTTGATGAGGATTTCCAC CCGGCG-3′. The amplified gene product was digested by *Bam*HI and *Hin*dIII (FastDigest, Thermo Fisher Scientific, Waltham, MA), cloned into the expression vector pET16b (Novagen, Darmstadt, Germany), and transformed into *E. coli* DH5α by heat shock transformation. All plasmids are listed in Table 1. The 6 × His-tagged proteins were expressed in *E. coli* BL21(DE3) (Novagen) after induction with 1 mM IPTG. Expressed proteins were purified on Ni-NTA resin columns (GE Healthcare, Chicago, IL) as previously described (Paulsson et al., 2019). Transcomplementation of the transposon mutant PW2884 was done by amplifying *pba* with 5′-ACTATTCGGATCCATTAAAGAGGAGAAATTACACATGAG CATCACGAGGAC-3′ and 5′-AGTCATGACAAGCTTTCA TTCGCGCTTGATGAGGATTTCCA-3′, digested with *Bam*HI and *Hin*dIII, and cloned into the complementation plasmid pUCP22Not. The construct was transformed into *E. coli* top 10 by heat shock and subsequently into PW2884 by electroporation to construct PW2884 + *pba*. The DNA sequences of all bacterial constructs were verified by sequencing (MWG Eurofins, Ebersberg, Germany) and cross-checked against the PAO1 genome deposited at the *Pseudomonas* Genome database (Winsor et al., 2015).

### Production of Rabbit Polyclonal Antibodies

Two rabbits were immunized with recombinantly expressed Pba (200 μg) emulsified in complete Freund’s adjuvant (Difco, Becton Dickinson, Heidelberg, Germany). Equal booster doses were injected on days 18 and 36. Blood was drawn 3 weeks later. Immunoblots were performed to ensure that each antiserum reacted with the expected recombinantly expressed protein. The resulting polyclonal antibodies (pAbs) were purified with affinity chromatography.

### Protein Gels and Western Blots

Proteins were separated by electrophoresis using NuPAGE Bis-Tris 4–12% gradient sodium dodecyl sulfate (SDS) polyacrylamide gels (Life Technologies, Carlsbad, CA). The gels were visualized by silver staining. Parallel gels were prepared and proteins were transferred from one gel onto a 0.45-μm Immobilon-P polyvinylidene fluoride (PVDF) membrane (Millipore, Darmstadt, Germany). Membranes were blocked with phosphate-buffered saline (PBS)–5% milk and washed with PBS–0.05% Tween-20 (PBS-T). After incubation with rabbit anti-Pba pAbs, the presence of Pba was detected using horseradish peroxidase (HRP)-conjugated anti-rabbit pAbs (Dako, Glostrup, Denmark) and ECL Western blotting substrate (Pierce, Rockford, IL) and visualized in ChemiDoc XRS + (BioRad, Hercules, CA).

### Purification of PG

The PG purification protocol was modified from Kühner et al. (2014).Briefly, bacteria were cultured overnight, washed in PBS, and resuspended in 1 ml of 1 M NaCl at OD_600_ = 20. The suspension was incubated at 99°C with 600 rpm shaking for 40 min. After centrifugation at 14,000 × *g* for 10 min, the pellets were washed and resuspended in water. Cell suspensions were thereafter sonicated three times for 1 min. The samples were pelleted and resuspended in 100 Tris–HCl, pH 6.8, with DNaseI (Sigma-Aldrich, St. Louis, MO) and RNase A (Sigma-Aldrich) before incubation at 37°C for 40 min. After pelleting, proteinase K (Sigma-Aldrich) was added, followed by incubation at 37°C for 60 min. Finally, enzymes were inactivated at 99°C and the PG saccules were washed in ddH_2_O and resuspended in 500 μl ddH_2_O. To break cross-linked sacculi, the samples were further sonicated for 10 s in three repetitions and finally stored at -20°C until use.

### Enzyme-Linked Immunosorbent Assay

Purified PGs were diluted in 1:100 coating buffer (100 mM Tris–HCl, pH 9.0) and coated in 96-well microtiter plates (Nunc PolySorp, Thermo Fisher Scientific). The plates were washed with PBS and blocked with PBS–2.5% bovine serum albumin (BSA). Recombinant Pba was added to the wells and incubated for 1 h at room temperature. In competitive inhibition assays, PGs were added to the wells together with Pba. Unbound Pba was removed by washing with PBS-T. HRP-conjugated anti-His pAbs (Abcam, Cambridge, United Kingdom) were added in PBS–2.5% BSA and incubated for 1 h before further PBS-T washes and development with 20 mM tetramethylbenzidine and 0.1 M potassium citrate. Finally, the reactions were stopped with 1 M H_2_SO_4_ and the absorbances read at 450 nm (Sunrise Tecan, Männedorf, Switzerland). The *P. aeruginosa* OMP OprG was used as a negative control.

### Sepharose Bead-Based ELISA

CNBr-activated agarose beads (Sepharose 4B, GE Healthcare) were rehydrated and washed according to the manufacturer’s instructions. Tri-DAP and Tri-LYS (InvivoGen, Toulouse, France) were diluted to 0.5 mg/ml in coupling buffer (0.1 M NaHCO_3_, pH 8.3, containing 0.5 M NaCl) and added to the beads at 4°C for 16 h. Excess ligands were washed away from the beads with coupling buffer. The Tri-LYS or Tri-DAP coupled beads were blocked for 2 h with blocking buffer (0.1 M Tris–HCl, pH 8.3, containing 0.5 M NaCl) and washed three times by alternate use of coupling and blocking buffer before resuspending them into PBS. The beads were incubated in PBS containing 1% milk and 0.05% Tween-20 (PMT) and transferred to 96-well V-bottom microtiter plates. His-tagged Pba or OprG as the control in PMT (Paulsson et al., 2019) was added to the beads and incubated for 1 h at 22°C on a shaker (500 rpm). The beads were sedimented by gentle centrifugation (20 × *g*, 2 min), the excess liquid carefully removed, and then washed with PMT. Bead-bound proteins were detected by HRP-coupled rabbit anti-6 × His pAbs (AbCam), followed by further washes before development as described for enzyme-linked immunosorbent assay (ELISA).

### Transmission Electron Microscopy of Bacterial Cells

For high-pressure freezing and freeze substitution protocol, bacteria were cultured in liquid broth and directly filled into 3-nm carriers, which subsequently were filled with hexadecane or 20% dextran solution. Bacterial cells were vitrified with Leica EM HMP100 and stored in liquid nitrogen until the freeze substitution process was initiated. Freeze substitution carousels were filled with 1% OsO_4_ and 0.5% uranyl acetate (from a 4% methanol stock solution) in acetone and cooled down in liquid nitrogen. Open carriers with bacteria were placed in the carousel and transferred to the freeze substitution equipment (Leica AFS2) sample chamber at −90°C. The freeze substitution protocol was modified from Bleck et al. (2010). Infiltration at −90°C lasted for 48 h before the temperature was raised with 5°C per hour until reaching −30°C. After 3 h, the staining solution was removed by washing three times and replaced with acetone. The sample chamber temperature was further raised 5°C per hour until 0°C. The bacterial cells were incubated with increasing epon concentrations in three steps, 1:2. 1:1, and 2:1 epon EMbed 812 (EMS, Hatfield, PA, United States), with each concentration lasting 2 h before exchange and finally 100% epon overnight. The epon resin was polymerized at 60°C for 48 h.

For conventional chemical fixation, bacteria were grown on chocolate agar and the cells fixed in 2.5% glutaraldehyde in 0.1 M sodium cacodylate overnight, followed by rinsing in 0.1 M sodium cacodylate for 15 min and post-fixation in 1% osmium tetroxide for 2 h. The cells were dehydrated in a graded ethanol series for 2 h, and the residual water was removed with propylene oxide for 30 min prior to embedding in Spurr’s resin (EMS) overnight and polymerization at 65°C for 8 h.

Ultrathin sections (80 nm) were obtained on an ultramicrotome (Leica Microsystems, Wetzlar, Germany) with a 45° diamond knife (Diatome, Nidau, Switzerland), placed on formvar-coated nickel (chemical fixation) or copper (freeze protocol) grids and counterstained with uranyl acetate and lead citrate. The samples were examined with a CM120 Biotwin electron microscope operating at 120 kV at ×7,400 and ×17,500 magnifications (Philips, Eindhoven, Netherlands). Micrographs were recorded with a Cantega G2 (Olympus SIS, Münster, Germany) 2K-pixel charge-coupled device camera using the iTEM software. Alternatively, the samples were examined with a JEOL 1230 electron microscope operating at 80 kV at ×50,000 and ×80,000 magnifications. The micrographs were recorded with Gatan 1K- or 4K-pixel charge-coupled device camera using the Digital Micrograph software.

### Isolation and Analysis of OMVs

Outer membrane vesicles were isolated according to the method described by Rosen *et al.*, with an additional size separation step as previously described (Rosen et al., 1995; Paulsson et al., 2018). Briefly, *P. aeruginosa* was grown shaken overnight at 37°C in Jensen media. The cells were removed by centrifugation and the collected supernatant was filtered through 0.45-μm pore filters (Sartorius, Göttingen, Germany). The filtered supernatant was further concentrated using a Vivaflow 200 Crossflow Cassette [polyethersulfone membrane (PES) with 100,000 molecular weight cutoff (MWCO); Sartorius, Germany] to retain any protein complex structures larger than 100 kDa. The OMVs in the concentrated supernatant were pelleted by ultracentrifugation at 100,000 × *g*. The pelleted OMVs were purified through density gradient separation in Histodenz (Sigma-Aldrich) and resuspension in PBS. Particle concentrations were determined by nanoparticle tracking analysis (NTA) using NanoSight LM10 (Malvern, Malvern, United Kingdom). The OMV content of each preparation with a protein concentration between 5 and 20 mg/ml was confirmed by transmission electron micrographs (TEMs). For negative staining, 3.5 μl of vesicles was adsorbed for 2 min onto glow-discharged formvar and carbon-coated copper grids, washed in H_2_O, and immediately negatively stained in 1.5% uranyl acetate solution for 30 s. Negative-stained samples were examined and recorded by TEM as described above.

### Flow Cell and Crystal Violet Biofilm Analysis

The green fluorescent protein (GFP)-encoding plasmid pMRP9 was transformed into *P. aeruginosa* PAO1 and mutants, and biofilms were grown as described by Davies et al. (1998) and Crusz et al. (2012). M9 minimal medium was continuously injected into a flow chamber with 10% (*v*/*v*) phosphate buffer (pH 6.7) supplemented with 0.3 mM glucose. The optical densities (OD_600_) of the overnight cultures were adjusted to OD_600_ = 0.01 before inoculation of the chambers using a 27-gauge needle and syringe. The chambers were stationary for 1 h before the pump was started. A laminar flow of 3 ml/h was maintained for the experiment’s duration with a Watson-Marlow 205S/CA pump (Watson-Marlow, Wilmington, MA). Biofilms were observed as *z*-stacks (1 μm steps) with a Zeiss Imager Z2 LSM 710 CLSM confocal microscope and the software Zeiss Zen 2010 v. 6.0 (Zeiss, Jena, Germany). Three-dimensional projections were produced in Imaris 8.2 (Bitplane, Zurich, Switzerland), and biomasses were quantified as pixels^3^ using FIJI/ImageJ with the plugin Voxel Counter (National Institutes of Health, Bethesda, MA).

For crystal violet experiments, overnight cultures in LB were pelleted and diluted to OD_600_ = 0.05. Fifty microliters of this suspension was added to 150 μl LB in sterile polystyrene 96-well microplates and incubated at 37°C for 16 h, still or shaking at 140 rpm as indicated. The liquid suspension was discarded and the plate was carefully washed twice with PBS before the addition of 200 μl of 0.2% crystal violet. The plate was washed three times with dH_2_O after 30 min incubation and then dried at 48°C. The biofilm was released with 33% acetic acid, 100 μl was transferred to a clean microplate, and the absorption measured at 540 nm.

### Statistical Analyses

Statistical differences were analyzed using Prism 8.2 (GraphPad Software, La Jolla, CA). Significance was calculated with two-tailed *t*-tests. For all experiments, *p* ≤ 0.05 were considered statistically significant.

### Ethics

Experiments utilizing animals were approved by the Swedish Board of Agriculture (Lund and Malmö Tingsrätt dnr. 4438/2017). All experiments were performed in accordance with the relevant guidelines and regulations set up by this institution.

## Results

### Pba Is Expressed in Clinical Strains and Localized in the Outer Membrane

In *P. aeruginosa*, Pba is expressed and translated from gene locus PA1041 as a 210-amino acid polypeptide, forming a 21.7-kDa lipoprotein with a predicted localization in the outer membrane based on the topology prediction software PSORTb V3.0. The Pba polypeptide contains a lipoprotein signal peptide [amino acids (aa) 1–19], followed by a short domain (aa 20–31) with unknown function and, finally, an OmpA domain (aa 32–210). The amino acid alignments of the full Pba sequence with selected OmpA family proteins are presented in Supplementary Figure 1. This alignment reveals a low identity (11.8–20.6%). However, the PG-binding domain (aa 138–203) shows a 68.2% identity with the corresponding part of OprF. The predicted tertiary structure of Pba has a pocket formed by three alpha helices and beta sheets in which *meso*-diaminopimelic acid (mDAP) is predicted to bind, and hence is the attachment point to PGs (Supplementary Figure 2). The previously suggested amino acids responsible for mDAP binding (aspartic acid at position 142 and arginine at 157) are conserved in Pba (Park et al., 2012).

**FIGURE 1:**
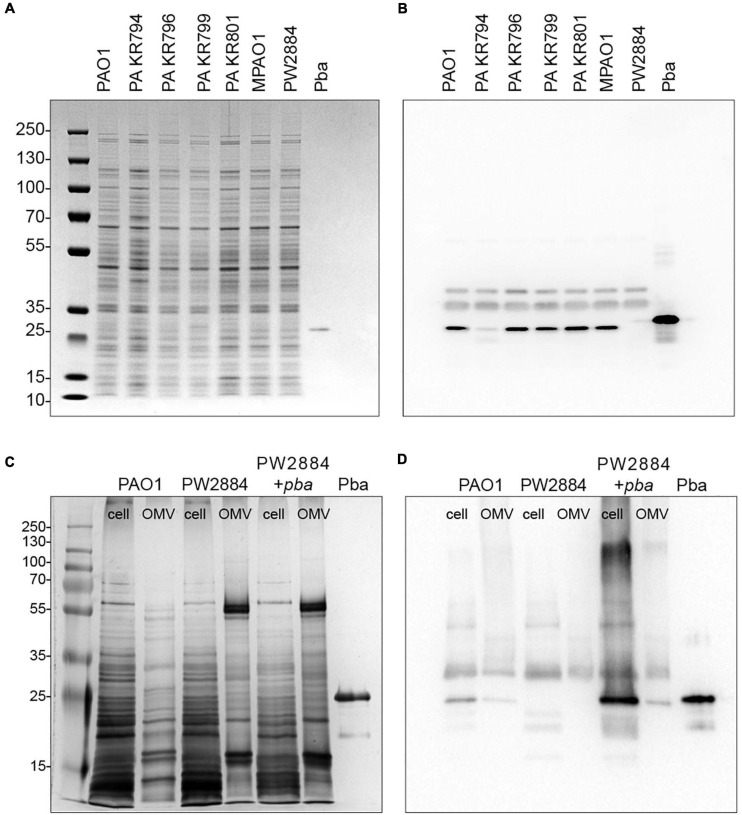
Peptidoglycan-binding anchor (Pba) is expressed by clinical strains and is absent in the *Pseudomonas aeruginosa* PW2884 mutant. Pba expressions of the reference strain PAO1, four clinical *P. aeruginosa* strains, the mutant PW2884 with a transposon insert in *pba*, and the corresponding wild-type strain (MPAO1). Bacterial outer membrane proteins (OMPs) and recombinantly expressed Pba were separated on SDS-PAGE and silver stained **(A)**. Pba was detected in western blot **(B)** from a corresponding gel with rabbit anti-Pba pAbs. PAO1, transposon mutant PW2884, and a transcomplemented strain (PW2884 + *pba*) were further analyzed for Pba content from whole cell lysates and purified OMVs in silver-stained SDS-PAGE **(C)** and Western blot **(D)**.

In order to verify Pba expression at the protein level and its putative presence in the OM, we isolated OM fractions and subjected the preparations to Western blotting followed by detection with rabbit anti-Pba pAbs. All four tested clinical strains carried Pba in the outer membrane, although for one strain (*P. aeruginosa* KR794), the band was fainter than in the other strains (Figures 1A,B), indicating a potentially decreased protein expression in *P. aeruginosa* KR794. We also verified that *P. aeruginosa* PW2884 with a mutated *pba* from a transposon library did not produce any Pba. This fact was further confirmed in cell lysates and purified OMV preparations from *P. aeruginosa* PAO1, PW2884, and the transcomplemented strain PW2884 + *pba*. Cell lysates and OMVs from the *P. aeruginosa* wild type and transcomplemented mutants both contained Pba (Figures 1C,D).

### Pba Binds to PG From Different Species by Attaching to the Pentapeptide

**FIGURE 2:**
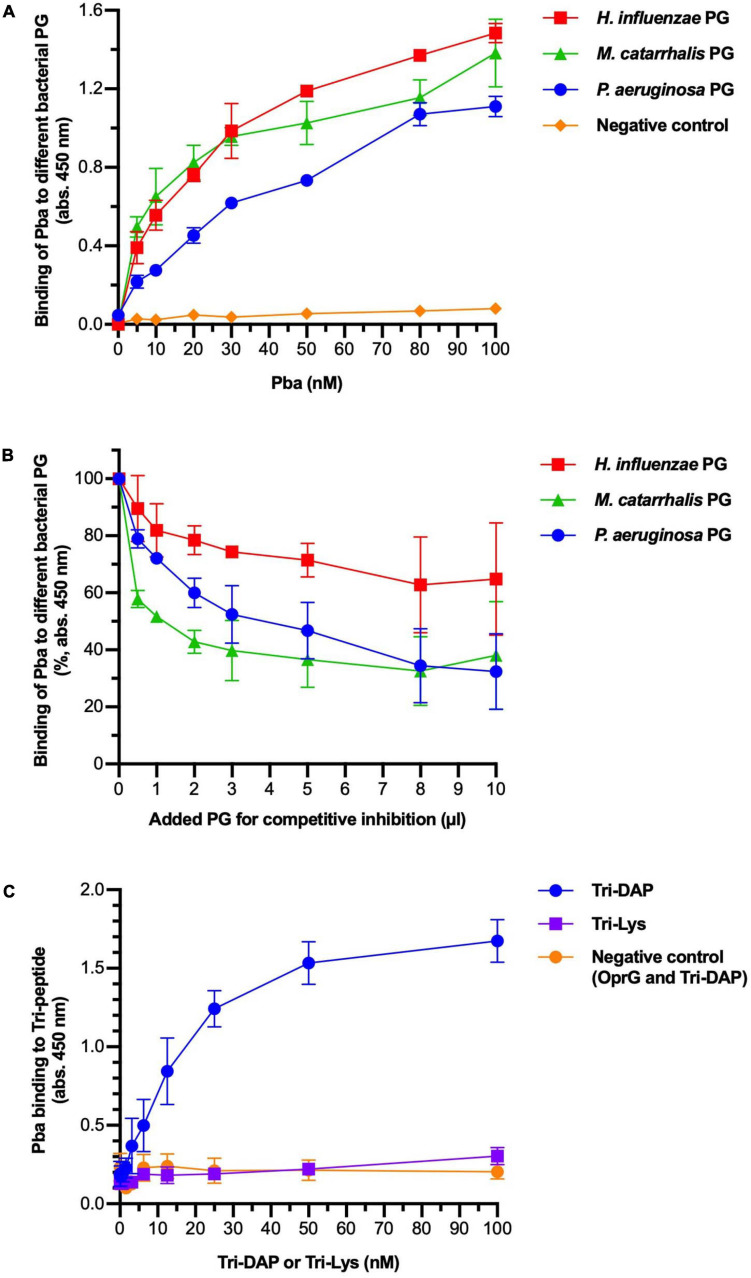
Peptidoglycan-binding anchor (Pba) binds to diaminopimelic acid (DAP) in the peptidoglycan (PG) peptide chain. The interactions between Pba and PG or PG components were analyzed by ELISA. **(A)** Peptidoglycans from three different Gram-negative bacterial species (*Pseudomonas aeruginosa*, *Haemophilus influenzae*, and *Moraxella catarrhalis*) were extracted and immobilized in 96-well plates. Recombinantly expressed 6 × His-tagged Pba or OprG (negative control) was added at increasing concentrations. Bound ligands were detected by HRP-conjugated anti-6 × His polyclonal antibodies (pAbs). **(B)** In a competitive inhibition ELISA, the PG was coated to the wells as above. Pba (50 nM) was preincubated with soluble PG at increasing concentrations before the addition of the mixture to the wells. Since the molarity of the PG sacculi could not be reliably determined, the volume of PG used for preincubation is indicated on the *x*-axis. Data are presented as absorption at 450 nm of initial value. To detect the binding of Pba to DAP, Tri-DAP (Ala-Glu-DAP), or Tri-LYS (Ala-Glu-Lys) was covalently bound to Sepharose beads, followed by a bead-based ELISA with Pba or OprG (negative control) as ligand **(C)**. The mean and standard deviations are plotted in all graphs, with data from three independent experiments.

Several OmpA family proteins have been reported to bind to PG. We assessed PG sacculi binding of Pba with ELISA (Figures 2A,B). Peptidoglycan sacculi were extracted from the bacterial cells of three different proteobacteria that share similar PG structures: *P. aeruginosa*, *H. influenzae*, and *Moraxella catarrhalis*. Purified PGs were immobilized in microtiter plates and Pba was added at increasing concentrations. Pba bound PG from *P. aeruginosa* PAO1, as well as PG from *H. influenzae* and *M. catarrhalis*, in a dose-dependent manner. This were expected results since the conserved PG-binding domain is present in the OmpA proteins in all three species. Our findings also illustrate that the Pba-dependent PG-binding capacity is not species specific but a highly conserved function of Gram-negative bacteria. The binding was partly inhibited by preincubating and saturating Pba with PGs from any of the Gram-negative bacterial species. Binding was also observed between Pba and PG sacculi with biolayer interferometry, but the dissociation constants could not be calculated due to the imprecise molarity determination caused by the heterogeneity in PG sacculi size (Supplementary Figure 3).

To specify binding to the pentapeptide portion of PG, the tripeptides L-Ala-γ-D-Glu-mDAP (Tri-DAP) and L-Ala-γ-D-Glu-Lys (Tri-Lys) were coupled to Sepharose beads that were further used in ELISA (Figure 2C). While Tri-DAP is part of the pentapeptide of Gram-negative PG, Tri-Lys belongs to the pentapeptide of Gram-positive PG and hence was considered as a negative control. The experiment revealed that Pba specifically bound to mDAP in a dose-dependent manner, and the Pba-dependent mDAP binding was saturated at higher ligand concentrations. Importantly, no detectable binding was found between the negative controls (Pba/Tri-Lys and OprG/Tri-DAP).

### Periplasm Expansion Occurs in the Poles of Pba Mutants

**FIGURE 3:**
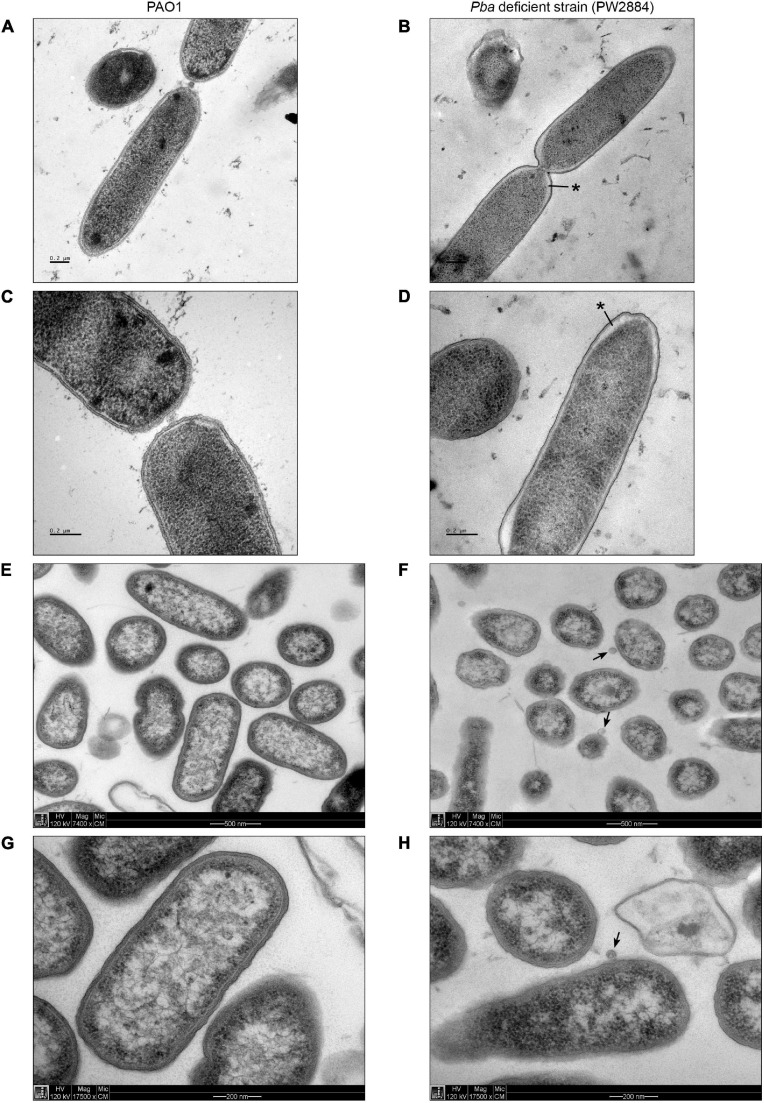
Peptidoglycan-binding anchor (Pba)-deficient *Pseudomonas aeruginosa* are morphologically different and shed outer membrane vesicles. Transmission electron micrographs of wild-type *P. aeruginosa* (PAO1) **(A,C,E,G)** and the corresponding mutated strain with a transposon ISlacZ/hah insert in *pba* (PW2884) **(B,D,F,H)**. Bacterial cells were fixated from liquid cultures with high-pressure freezing (HPF) **(A–D)** and from agar plates with chemfix **(E–H)**. A widened periplasmic space is depicted at the poles of Pba-deficient cells fixated with HPF (*black asterisks*), but not with chemfix. Outer membrane vesicles are present only in chemfixed Pba-deficient cells (*black arrows*). Pictures were recorded at ×7,400 **(A,B,E,F)** or ×17,500 **(C,D,G,H)** magnification.

To investigate the morphology of Pba-deficient *P. aeruginosa* PW2884 with TEM, we prepared cells using high-pressure freezing as well as chemical fixation. Pba-deficient cells were similarly sized and shaped as their wild-type counterparts. High-pressure freezing revealed a marked clearing under the cell envelope adjacent to the poles. The periplasm was widened to approximately 40 nm as compared to the 10 nm seen in the wild-type strain. A similar finding was seen in most *P. aeruginosa* PW2884 cells devoid of Pba, including bacteria observed during division (Figures 3A–D). Despite this changed phenotype with severe morphological alterations, the transposon mutant *P. aeruginosa* PW2884 had a growth rate that was comparable to that of the wild-type strain (Supplementary Figure 4). In addition, these micrographs revealed the chemically fixed *P. aeruginosa* PW2884 mutant from agar plates was surrounded by OMVs (Figures 3F,H).

### Pba Deficiency Causes Increased OMV Formation

**FIGURE 4:**
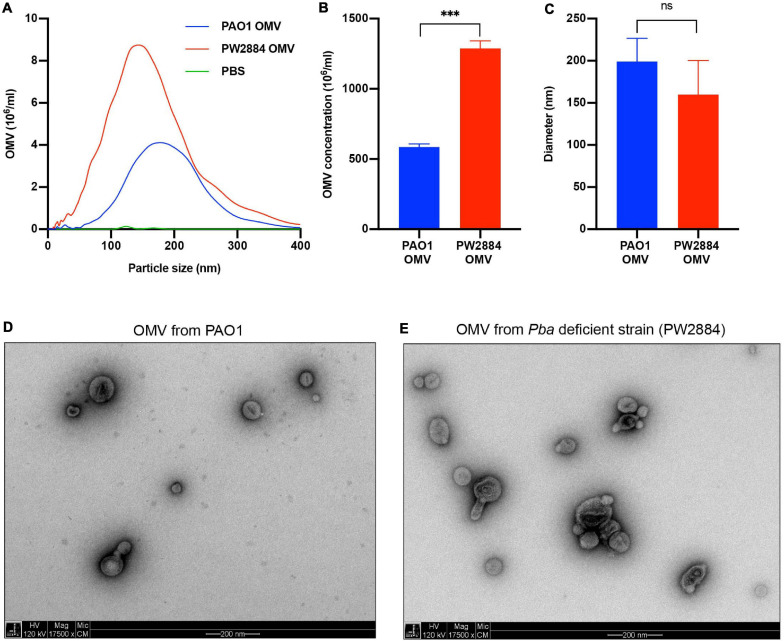
Peptidoglycan-binding anchor (Pba)-deficient *Pseudomonas aeruginosa* releases more outer membrane vesicles (OMVs). Nanoparticle tracking analysis revealed that the Pba-deficient mutant PW2884 released more OMVs than did the wild-type strain PAO1. As seen in the frequency plot **(A)**, all particle sizes were overrepresented in PW2884 compared to PAO1 and technical control (PBS). Significantly higher concentrations of OMVs were observed from the mutated strain **(B)**, but the average diameter of the particles was unaltered **(C)**. Transmission electron micrographs (TEM) confirmed that the OMVs from each strain were similar in size and shape and that the sample preparations were free from other cellular products that could bias the analysis **(D,E)**. ***P < 0.001, ns = not significant.

Outer membrane vesicles are released from the bacterial OM, and TEM pictures suggested increased blebbing when *P. aeruginosa* was devoid of Pba. To quantify the OMV release, the Pba-deficient mutated strain *P. aeruginosa* PW2884 and the wild-type counterpart were cultured overnight followed by purification of OMVs from the supernatant. OMVs sized up to 400 nm were analyzed using NTA (Figures 4A–C). The mean OMV concentration in the final preparations was significantly higher in the mutated *P. aeruginosa* strain PW2884 than in the wild-type strain (1.29 × 10^9^ OMVs/ml *vs*. 0.58 × 10^9^ OMVs/ml, *p* < 0.001). There was no marked OMV mean size difference between the strains. As indicated by the lower mode than mean OMV size [wild type (WT) mode = 178 nm, mean = 199 nm; PW2884 mode = 144 nm, mean = 160 nm), the size distributions were partially shifted to the right, indicating that a portion of the vesicles aggregated. The unaltered morphology of the OMVs was verified by TEM when OMVs from matching preparations were analyzed (Figures 4D,E).

### *Pseudomonas aeruginosa* Devoid of Pba Form Unorganized and Thick Biofilms

**FIGURE 5:**
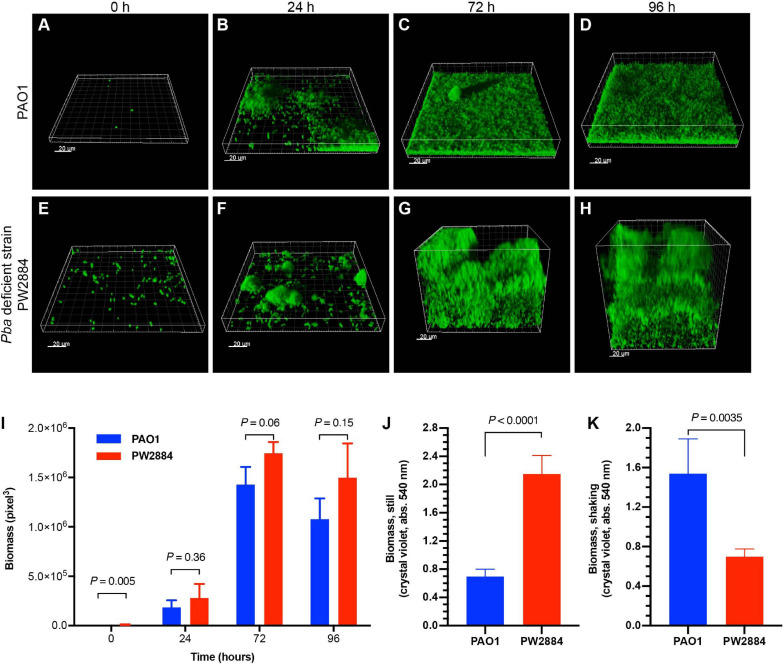
Peptidoglycan-binding anchor (Pba)-deficient *Pseudomonas aeruginosa* form thick and unstructured low-density biofilms. Flow cell models of the wild type PAO1 **(A–D)** and the Pba-deficient PW2884 mutant **(E–H)**, both carrying a green fluorescent protein (GFP) expression plasmid. Pictures were captured by confocal microscopy at start and after 24, 72, and 96 h. Instead of the dense biofilm formed by the wild-type strain, the biofilm from the Pba-deficient strain occupied a larger volume, but with only slightly larger biomass as measured by three-dimensional pixels **(I)**. In parallel, biofilms were cultured in 96-well plates in rich media and stained by crystal violet **(J,K)**. Biofilms from the mutated strain formed higher biomass in plates that were cultured stationary, whereas shaking led to the detection of lower biomass than in the wild-type strain **(J,K)**. Statistical analysis was made with *t*-test and *p*-values are indicated on the graphs.

To examine the effect of Pba on biofilm formation, GFP-tagged wild-type *P. aeruginosa* PAO1 and Pba-deficient mutated PW2884 were inoculated in a continuous flow cell system. After attachment to the flow cell’s bottom plate, the bacteria were cultured under continuous media flow for 96 h. Three-dimensional confocal microscopy images were acquired at the start of the experiment and after each 24 h (Figures 5A–H). The wild-type strain formed a dense structured carpet of attached bacteria with single mushroom-like structures. In contrast, the Pba-deficient *P. aeruginosa* PW2884 formed a thicker biofilm than the biofilm formed by the wild-type counterpart. Although more PW2884 cells were attached at the first time point (biomass = 318 *vs*. 9,792 pixels^3^, *p* = 0.005), the biomass of the final biofilm was only slightly higher in the mutant strain devoid of Pba, indicating looser attachment between the bacterial cells (Figure 5I). In parallel experiments, bacteria were grown in microtiter plates that were incubated with or without shaking of the plates. In this experiment, the biomass was measured using crystal violet (Figures 5J,K). Here, *P. aeruginosa* PW2884 formed more biofilms without shaking, but less when the plate was shaken during incubation. These two experiments indicated that, *P. aeruginosa* PW2884 devoid of Pba in the OM formed a thick, but non-dense and fragile biofilm.

In separate experiments, adhesion to glass slides with or without prior protein coating was examined (Supplementary Figure 5). No difference in adhesive properties could be found between the wild-type and mutated strains in these experiments. Hence, the altered biofilms observed in flow cells were caused by the poor structure in the biofilm itself and not by impaired adhesion to the flow cell or microtiter plate.

## Discussion

In this study, we present evidence that Pba is a lipoprotein in the outer membrane of *P. aeruginosa* encoded by the gene locus PA1041. It belongs to the OmpA protein family and plays important roles in cellular morphology, OMV release, and biofilm formation. In addition, we confirmed that mDAP in the PG pentapeptide is the inner attachment point for Pba, by which it tethers the outer membrane to the PG.

The large *P. aeruginosa* genome contains several other genes that code for OmpA family proteins including OprF, OrpL, and PA0833. Hence, it is remarkable that the present and several previous studies report extensive phenotypic changes when a single gene is depleted. Functional overlap between these proteins may explain the unaltered growth rate in the Pba-deficient mutant *P. aeruginosa* PW2884.

Pba was found in outer membrane preparations. The *E. coli* OmpA protein PAL has been reported to have dual orientations (Michel et al., 2015). This finding suggests that an exterior segment of the protein protrudes from the outer membrane. It is not possible to determine the orientation of Pba from our results. Any additional function of the Pba N-terminal than membrane integration is yet unknown. The most abundant OmpA protein in *P. aeruginosa* is OprF (Chevalier et al., 2017). The membrane domain of OprF forms an aqueous channel, while the periplasmic part tethers to the PG. Mutated strains lacking the OprF C-terminal had reduced cell lengths and were sensitive to low osmolarity (Rawling et al., 1998). We observed that Pba mutants had an increased periplasmic space near the poles when preparing cells with the freeze substitution protocol in TEM, but no general alteration in cell shape was observed. Membrane artifacts are generally not observed when the freeze substitution protocols are used, and these micrographs should accurately depict the periplasm (Hunter and Beveridge, 2005). Previous reports found the cell wall of mutated *H. influenzae* lacking the OmpA family protein P6 to be distorted. The outer membrane appeared wrinkled and the electron microscopy pictures contained several OMVs (Murphy et al., 2006). Similarly, with TEM, we observed that the *P. aeruginosa* Pba mutants cultured on agar plates were surrounded by OMVs. When counted with nanoparticle tracking analysis, significantly more OMVs were released from the mutated cells than from the wild type. OMV release from OprF-deficient mutants has been reported to be increased 8-fold when measuring the phospholipid bilayer content and is suggested to be regulated by PQS (Wessel et al., 2013). Further studies are needed to clarify whether Pba mutants produce more OMVs because of loss of PG tethering or increase of PQS in the periplasm.

A novel bead-based ELISA was developed in which the tripeptides Tri-DAP and Tri-Lys were covalently linked to CNBr-activated agarose beads. The beads were sedimented through gentle centrifugation and the unbound protein in the supernatant was removed. Bound ligands were opsonized by HRP-linked 6 × His pAbs and could be detected using standard ELISA methods. The method accurately discriminated Tri-DAP binding Pba from negative controls. A challenge with the method was that the beads were easily removed while washing and great care had to be taken in these steps.

The properties of the biofilms formed by Pba-deficient mutants were dependent on the culture conditions. In flow cells, the biofilms were thick, but unstructured and of low density. When cultured in rich media in microtiter plates, Pba mutants formed biofilms with increased biomass, but if the plate was shaken, much less biofilm was formed by the mutated strain than the wild type. Similarly, OprF-deficient mutated strains formed biofilms with increased biomass when cultured in rich media in microplates without shaking and a thick biofilm in flow cells (Bouffartigues et al., 2015). We speculate that the unorganized structure and low density makes the biofilm of Pba-deficient mutants more fragile and that shaking may have caused the biofilm to collapse or detach.

A limitation of the present study is the lack of phenotypic analysis that includes the transcomplemented mutant (PW2884 + *pba*). This strain overexpressed Pba, and while this did not affect the growth rate, it negatively interfered with the biofilm and OMV analysis.

In this study, we have expressed and characterized the previously hypothetical lipoprotein PA1041 as a PG-binding lipoprotein in the OM of *P. aeruginosa*, and we suggest the annotation “peptidoglycan binding anchor” (Pba). We have observed that it is expressed in clinical strains and we have confirmed the PG-binding property. Several phenotypic alterations in the mutated strains have been observed, and although these are intuitively explained by loss of tethering to the PG layer, further studies are needed to elucidate the involvement of PQS, stress responses, or other transcriptional responses. Importantly, outer membrane localization enables the use of Pba as a vaccine target, and Pba could, just like other OmpA family proteins, be evaluated for its immunogenic potential.

## Data Availability Statement

The raw data supporting the conclusions of this article will be made available by the authors, without undue reservation.

## Ethics Statement

Experiments utilizing animals were approved by the Swedish Board of Agriculture (Lund and Malmö Tingsrätt dnr. 4438/2017). All experiments were performed in accordance with relevant guidelines and regulations set up by this institution.

## Author Contributions

MP, Y-CS, and KR planned the study. KK and TB performed the flow cell experiments. LS did electron microscopy. BS extracted PG and did the ELISA experiments. MP or technical staff employed by KR did all other laboratory work. MP drafted the manuscript. All authors reviewed the manuscript and agreed to be accountable for the content of the work.

## Conflict of Interest

The authors declare that the research was conducted in the absence of any commercial or financial relationships that could be construed as a potential conflict of interest.
