# Supplementary material for: Peptidoglycan-Binding Anchor Is a Pseudomonas aeruginosa OmpA Family Lipoprotein With Importance for Outer Membrane Vesicles, Biofilms, and the Periplasmic Shape
Source: Front Microbiol. 2021 Feb 25;12:639582. doi: 10.3389/fmicb.2021.639582 (PMC7947798; doi:10.3389/fmicb.2021.639582)
Supplement: Supplementary file 1 [file Table_1.DOCX]

Supplementary Material

# Supplementary Figures

| Reference sequence (1): Pba  Identities normalised by aligned length.  Colored by: identity |
| --- |
| cov pid  **1** **[ . . . . : . . .** **80**  1 Pba 100.0% 100.0% **--------------------------------------------------------------------------------**  2 OprF 86.7% 20.6% **MKLKNTLGVVIGSLVAASAMNAFAQGQNSVEIEAFGKR-YFTDSVRN-------------MKNADLYGGSIGYFLTDDVE**  3 AbOmpA 82.9% 13.8% **MKLSRI---ALATMLVAAPLAAAN---AGVTVTPLLLGYTFQDSQHNNGGKDGNLTNGPELQDDLFVGAALGIELTPWLG**  4 EcPal 81.4% 18.7% **--------------------------------------------------------------------------------**  5 HiP6 71.9% 17.4% **--------------------------------------------------------------------------------**  6 KpOmpA 74.8% 11.8% **MK-------------AIFVLNAAPKDNTWYAGGKLGWSQYHDTGF---YGNGFQNNNGPTRNDQLGAGAFGGYQVNPYLG**  consensus/100% **................................................................................**  consensus/90% **................................................................................**  consensus/80% **................................................................................**  consensus/70% **................................................................................**  cov pid  **81**  **. 1 . . . . : .** **160**  1 Pba 100.0% 100.0% **--------------------------------------------------------------------------------**  2 OprF 86.7% 20.6% **LALSYGEYHDV--RGTYET-------GNKKVHGNLTSLDAIYHFGTPGVGLRPYVSAGLAHQ------NI--TNINSDSQ**  3 AbOmpA 82.9% 13.8% **FE---AEYNQV--KGDVDGASAGAEYKQKQINGNFYVTSDLI-TKNYDSKIKPYVLLGAGHY------KYDFDGVNRGTR**  4 EcPal 81.4% 18.7% **--------------------------------------------------------------------------------**  5 HiP6 71.9% 17.4% **--------------------------------------------------------------------------------**  6 KpOmpA 74.8% 11.8% **FEMGYDWLGRMAYKGSVDNGAFKAQGVQLTAKLGYPITDD----------LDIYTRLGGMVWRADSKGNYASTGVSRSEH**  consensus/100% **................................................................................**  consensus/90% **................................................................................**  consensus/80% **................................................................................**  consensus/70% **................................................................................**  cov pid **161**  **. . . 2 . . . .** **240**  1 Pba 100.0% 100.0% **-----MSIT--RTALPLI-----LVSGLLTGCAGLQKSDWPTCAAVGGVTGAGLGAIESSSWAGWG--ALIGGGVGAAYC**  2 OprF 86.7% 20.6% **GRQQ-MTMANIGAGLKYYFTENFFAKASLDGQYGLEKR-------------------DNGHQGEWM--AGLGV--G--FN**  3 AbOmpA 82.9% 13.8% **GTSEEGTLGNAGVGAFWRLNDALSLRTEARA-------------------------TYNADEEFWNYTALAGLNV-----**  4 EcPal 81.4% 18.7% **-----MQL-------------NKVLKGLMIAL---PVMAIAACSSN------KNASND-GSEGMLG--AGTGMDA-----**  5 HiP6 71.9% 17.4% **-------M-------------NKFVKSLLVAG---SVAALAACSSS------NNDAAGNGAAQTFG--------------**  6 KpOmpA 74.8% 11.8% **DTGV---SPVFAGGVEWAVTRDI--------------------------------------------------ATRLEYQ**  consensus/100% **................................................................................**  consensus/90% **................................................................................**  consensus/80% **.......h.............sh.hps.h.u............................ut.t.h...............**  consensus/70% **.......h.............sh.hps.h.u............................ut.t.h...............**  cov pid **241**  **: . . . . 3 . .** **320**  1 Pba 100.0% 100.0% **WVHGAGEQ--VAPPPPQPVEEV--------------------------APPPPVVKEETIVVRDLHFAFDSSKVDAADSE**  2 OprF 86.7% 20.6% **F----GGS--KAAPAPEPVADVCSDSDNDGVCDNVDKCPDTPANVTVDANGCPAVAEVVRVQLDVKFDFDKSKVKENSYA**  3 AbOmpA 82.9% 13.8% **-VL--GGH--LKP--AAPVVEV------------------APVEPTPVAPQPQELTEDLNMELRVFFDTNKSNIKDQYKP**  4 EcPal 81.4% 18.7% **-NGGNGNM--SS------------------------------------EEQARLQMQQLQQNNIVYFDLDKYDIRSDFAQ**  5 HiP6 71.9% 17.4% **---G---Y---------------------------------------------SVADLQQRYNTVYFGFDKYDITGEYVQ**  6 KpOmpA 74.8% 11.8% **WVNNIGDAGTVGTRPDNGML-------SLGVSYRFGQEDAAPVVAPAPAPAPEVATKHFTLKSDVLFNFNKATLKPEGQQ**  consensus/100% **.......................................................hp.......lhFshsp.plp.t...**  consensus/90% **.......................................................hp.......lhFshsp.plp.t...**  consensus/80% **.....Gt....t....................................t..s..htc.hph..pVhFshsK.plcsp..t**  consensus/70% **.....Gt....t....................................t..s..htc.hph..pVhFshsK.plcsp..t**  cov pid **321**  **. . : . . . . 4** **400**  1 Pba 100.0% 100.0% **KLNGIAERLKGEAAS-TRLSITGHTDSVGSDAYNQKLSERRANAVANYLIDAGVPSSIIVGVQGLGESQPVADNKTRE--**  2 OprF 86.7% 20.6% **DIKNLADFMK-QYPS-TSTTVEGHTDSVGTDAYNQKLSERRANAVRDVLVNEYGVEGGRVNAVGYGESRPVADNATAE--**  3 AbOmpA 82.9% 13.8% **EIAKVAEKLS-EYPN-ATARIEGHTDNTGPRKLNERLSLARANSVKSALVNEYNVDASRLSTQGFAWDQPIADNKTKE--**  4 EcPal 81.4% 18.7% **MLDAHANFLR-SNPS-YKVTVEGHADERGTPEYNISLGERRANAVKMYLQGKG-VSADQISIVSYGKEKPAVLGHDEA--**  5 HiP6 71.9% 17.4% **ILDAHAAYLN-ATPA-AKVLVEGNTDERGTPEYNIALGQRRADAVKGYLAGKG-VDAGKLGTVSYGEEKPAVLGHDEA--**  6 KpOmpA 74.8% 11.8% **ALDQLYTQLSNMDPKDGSAVVLGYTDRIGSEAYNQQLSEKRAQSVVDYLVAKG-IPAGKISARGMGESNPVTGNTCDNVK**  consensus/100% **.ltthht.hp...st.hph.l.G.sDphGs.thN.tLu.tRApuVh.hL.sth...u..lsh.uhu.ppPhs.ststt..**  consensus/90% **.ltthht.hp...st.hph.l.G.sDphGs.thN.tLu.tRApuVh.hL.sth...u..lsh.uhu.ppPhs.ststt..**  consensus/80% **.lsthAphLp.t.Ps.spshlpGaTDphGo.tYN.pLup+RAsuVtshLhsch.lsusplus.uhGcppPss.spsct..**  consensus/70% **.lsthAphLp.t.Ps.spshlpGaTDphGo.tYN.pLup+RAsuVtshLhsch.lsusplus.uhGcppPss.spsct..**  cov pid **401**  **. . . ]** **440**  1 Pba 100.0% 100.0% **-------GRAENRRVEILIKRE------------------**  2 OprF 86.7% 20.6% **-------GRAINRRVEAEVEAEAK----------------**  3 AbOmpA 82.9% 13.8% **-------GRAMNRRVFATITGSRTVVVQPGQEAAAPAAAQ**  4 EcPal 81.4% 18.7% **-------AYSKNRRAVLVY---------------------**  5 HiP6 71.9% 17.4% **-------AYSKNRRAVLAY---------------------**  6 KpOmpA 74.8% 11.8% **ARAALIDCLAPDRRVEIEVKGYKEVVTQPQA---------**  consensus/100% **.......shu.sRRs.h.h.....................**  consensus/90% **.......shu.sRRs.h.h.....................**  consensus/80% **.......uhu.NRRs.h.h.....................**  consensus/70% **.......uhu.NRRs.h.h.....................** |

**Supplementary Figure 1.** Multiple amino acid sequence alignment of various Pba orthologoues including *P. aeruginosa* OprF, *Acinetobacter baumannii* OmpA (AbOmpA), *Escherichia coli* Pal (EcPal), *Haemophilus influenzae* P6 (HiP6), and, finally, *Klebsiella pneumoniae* OmpA (KpOmpA). Alignment was generated with Clustal O and formatted with Mview 1.63 at EMBL-EBI with colouring for amino acid identity (https://www.ebi.ac.uk/Tools/msa/mview/).


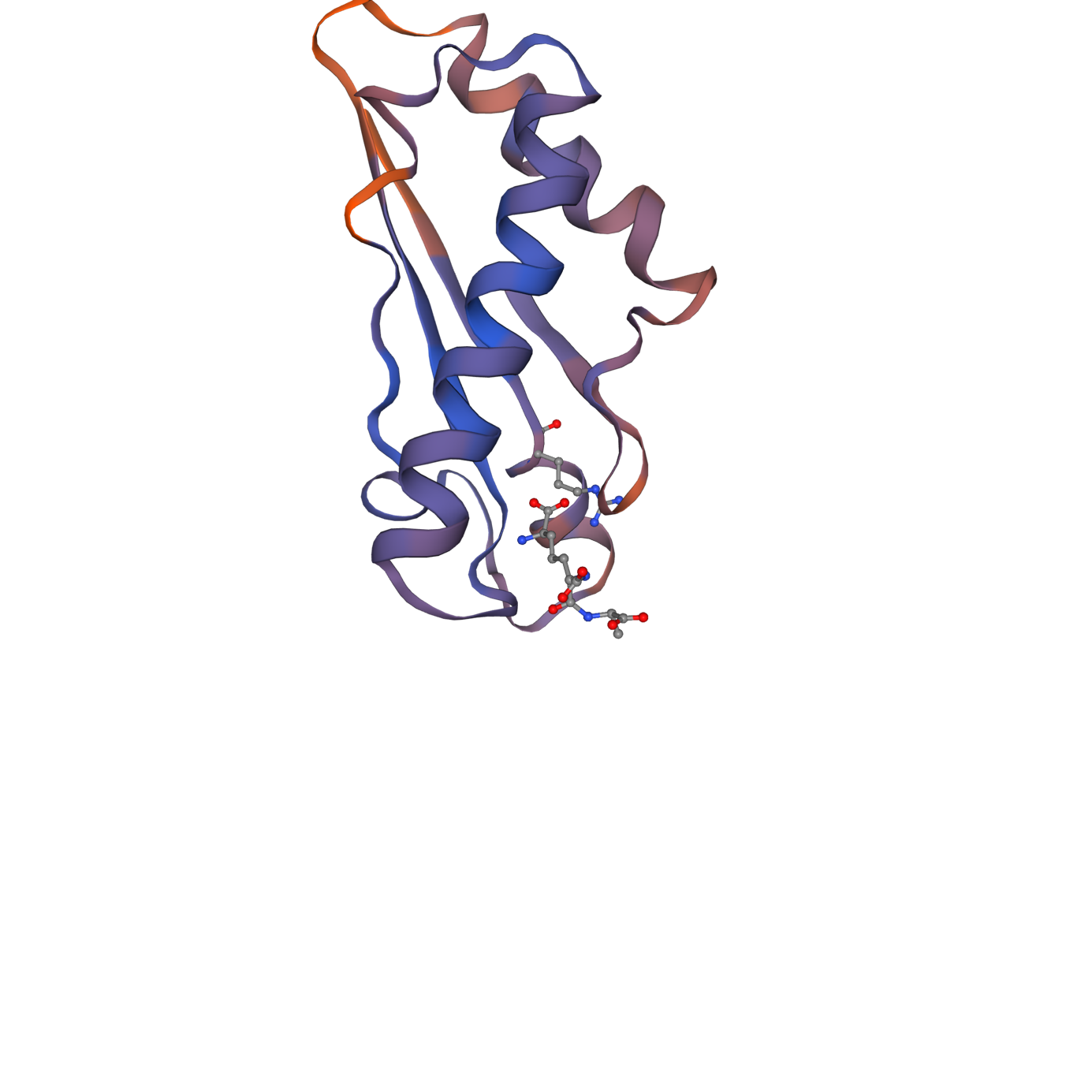


**Supplementary Figure 2.** Three-dimensional ribbon image model of Pba and TriDAP as a ligand. The model was based on the crystal structure of the OmpA domain of OprF. Three alpha-helixes and three beta sheets form a pocket in which mDAP is bound. Model generated by Swiss-model (<https://swissmodel.expasy.org>) for Pba (Q9I4T3) and 5u1h.1.A as template. Coloring represent quality estimate (QMEAN) of the protein structure model.

**Supplementary Figure 3.** A dose-dependent binding kinetics was observed with Pba and PG sacculi when analyzed by bio-layer interferometry (Octet Red96; Pall, Menlo Park, CA). Sensors were activated by submersion into 0.2 M 1-ethyl-3-(3-dimethylaminopropyl) carbodiimide and 0.05 M *N*-hydroxysulfosuccinimide. Pba in 10 mM sodium acetate pH 5 was immobilized on amine reactive (AR2G) sensors. Approximately 5 nm protein layer was built up prior to quenching with 2 M ethanolamine. Purified PGs were dialyzed in PBS and used for analysis of binding kinetics. One empty sensor and one Pba-bound sensor in PBS were used as controls. Data handling was done in the accompanying software (Octet Data acquisition and Data Analysis 8.1; Pall).

**Supplementary Figure 4.** Growth rate of mutated and wildtype strains were similar. Bacterial cells were cultured in 96-well plates in LB media at 37 °C. Absorption at 600 nm was measured 6 times per hour in a plate reader (Fluostar Omega, BMG Labtech, Ortenberg, Germany).

**Supplementary Figure 5.** Adhesion to protein coated glass slides. Wildtype PAO1 and PW2884 were added to protein-coated glass slides. Each slide was coated with 0.5 μg human plasma vitronectin and dried at 37 °C. Bacteria were grown in LB medium, washed and resuspended in PBS to OD_600_ = 0.5. The slide was submerged in the bacterial suspension and incubated at 37 °C for 1 h. Slides were rinsed after incubation and attached cells were gram-stained and counted in light microscopy. Adherent bacteria were automatically counted by the light microscopy image analysis software cellSens (Olympus) in 6 randomly selected regions of interest (ROI, red), in biological triplicates.
